# CLDN15 is a novel diagnostic marker for malignant pleural mesothelioma

**DOI:** 10.1038/s41598-021-91464-0

**Published:** 2021-06-15

**Authors:** Masayuki Watanabe, Tomohito Higashi, Kana Ozeki, Atsuko Y. Higashi, Kotaro Sugimoto, Hayato Mine, Hironori Takagi, Yuki Ozaki, Satoshi Muto, Naoyuki Okabe, Yuki Matsumura, Takeo Hasegawa, Yutaka Shio, Hiroyuki Suzuki, Hideki Chiba

**Affiliations:** 1 Department of Chest Surgery, Graduate School of Medicine, Fukushima Medical University, 1 Hikarigaoka, Fukushima, 960-1295 Japan; 2 Department of Basic Pathology, Graduate School of Medicine, Fukushima Medical University, 1 Hikarigaoka, Fukushima, 960-1295 Japan

**Keywords:** Diagnostic markers, Mesothelioma

## Abstract

Malignant mesothelioma is a cancer with a poor survival rate. It is difficult to diagnose mesotheliomas because they show a variety of histological patterns similar to those of various other cancers. However, since currently used positive markers for mesotheliomas may show false positives or false negatives, a novel mesothelial positive marker is required. In the present study, we screened 25 claudins and found that claudin-15 is expressed in the mesothelial cells. We made new rat anti-human claudin-15 (CLDN15) monoclonal antibodies that selectively recognize CLDN15, and investigated whether CLDN15 is a good positive marker for malignant pleural mesotheliomas (MPMs) using MPM tissue samples by immunohistochemistry and semi-quantification of the expression level using an immunoreactive score (IRS) method. Of 42 MPM samples, 83% were positive for CLDN15. The positive ratio was equal to or greater than other positive markers for MPMs including calretinin (81%), WT-1 (50%), and D2-40 (81%). In 50 lung adenocarcinoma sections, four cases were positive for CLDN15 and the specificity (92%) was comparable with other markers (90–100%). Notably, CLDN15 was rarely detected in 24 non-mesothelial tumors in the tissue microarray (12/327 cases). In conclusion, CLDN15 can be used in the clinical setting as a positive marker for MPM diagnosis.

## Introduction

Malignant mesothelioma is a cancer with a poor survival rate^1,2^. It originates from mesothelial cells that cover the outer layer of serous membranes, including the pleura, peritoneum, pericardium, and tunica vaginalis testis. Malignant pleural mesotheliomas (MPMs) account for 85.5% of the total malignant mesotheliomas^3^. Histologically, MPMs are divided into epithelioid, biphasic and sarcomatoid subtypes. MPMs are principally caused by exposure to asbestos, and it typically takes 30–40 years to develop a tumor after initial asbestos exposure^2–4^; however, its pathogenesis has yet been established^5^. In the majority of developed countries, asbestos production, handling and use are limited and asbestos consumption has fallen to negligible levels. However, dismantling of buildings containing asbestos is expected to increase in future, and asbestos use is unfortunately still not prohibited in some developing countries. Therefore, malignant mesothelioma will continue to represent a significant global health concern.

Symptoms of MPM include breathlessness, chest pain, fatigue, anorexia, and weight loss^6^ which are similar to those of lung adenocarcinoma. However, the treatment and prognosis are completely different. Treatment of MPMs mainly includes surgery, chemotherapy, and radiotherapy. Even if MPM patients receive these treatments, the median survival is limited to 9–12 months^7^. On the other hand, the survival rate of lung adenocarcinoma has been improving due to the development of targeted therapies and immunotherapeutic agents^8^.

It is difficult to diagnose mesotheliomas because they show various histological patterns similar to those of other cancers. In particular, it is important to distinguish MPMs from lung adenocarcinomas. The histology of epithelioid-type mesothelioma are often solid, tubulopapillary, and trabecular, which resembles that of lung adenocarcinomas. To discriminate MPMs from other tumors, immunohistochemical staining is required to confirm that at least two markers of mesothelial leneage are positive and at least two markers of epithelial lineage are negative^9^. Calretinin, WT-1 (Wilms' tumor 1), D2-40 (Podoplanin) and CK (Cytokeratin) 5/6 are clinically used as positive markers for epithelioid-type MPMs and carcinoembryonic antigen (CEA), claudin-4, thyroid transcription factor (TTF)-1, Napsin A, MOC31 and BerEP4 are used as negative markers. Although the sensitivity of Calretinin (nearly 100%), D2-40 (80–100%), and CK5/6 (75–100%) are high in the epithelioid-type MPMs, they are not exclusively specific to MPMs^10,11^. Calretinin and D2-40 are focally positive in 5–10% and about 15% of lung adenocarcinomas, respectively, and CK5/6 is positive in almost 100% of squamous carcinomas^10^. On the other hand, WT-1 has very high specificity, but its sensitivity is about 70–95% in the epithelioid-type MPMs^10^. In the sarcomatoid-type MPMs (and the sarcomatoid part of the biphasic MPMs), the sensitivity of these mesothelial markers are lower: Calretinin (50–60%), WT-1 (10–45%), D2-40 (75–90%) and CK5/6 (13–29%)^11^. Therefore, additional mesothelial positive markers with high sensitivity and specificity have been explored^12,13^.

Claudins (CLDNs) are major components of tight junctions, which seal the intercellular spaces between adjacent cells, such as epithelial and endothelial cells. They are four-transmembrane proteins with typically ~ 22-kDa molecular weight. The CLDN family comprises more than 24 members in human and mice, and specific combinations of CLDNs are expressed in normal epithelial tissues. In addition, CLDNs exhibit aberrant expression in a variety of cancer tissues^14–16^, some of which are used as diagnostic and/or prognostic markers for the cancer^17–19^. To date, only a few reports on the expression profile of CLDNs in mesothelial tissues exist^20^. It is also unknown which CLDN proteins are expressed in human malignant mesothelioma tissues. On the other hand, several lines of evidence indicate that the expression of *CLDN15* mRNA is overexpressed in the epithelioid subtype of MPMs^21–24^, which accounts for approximately 60% of MPM cases^25^.

Here, we investigated the expression and localization of CLDNs in normal mesothelial tissues and found that CLDN15 was the most abundantly expressed claudin in these tissues. Using a novel CLDN15-targeting monoclonal antibody (mAbs), we examined the expression of CLDN15 in human MPM tissue specimens and propose that CLDN15 can be a good positive marker for MPMs.

## Results

### Expression of claudin-15 in normal mesothelium in mice

**Figure 1:**
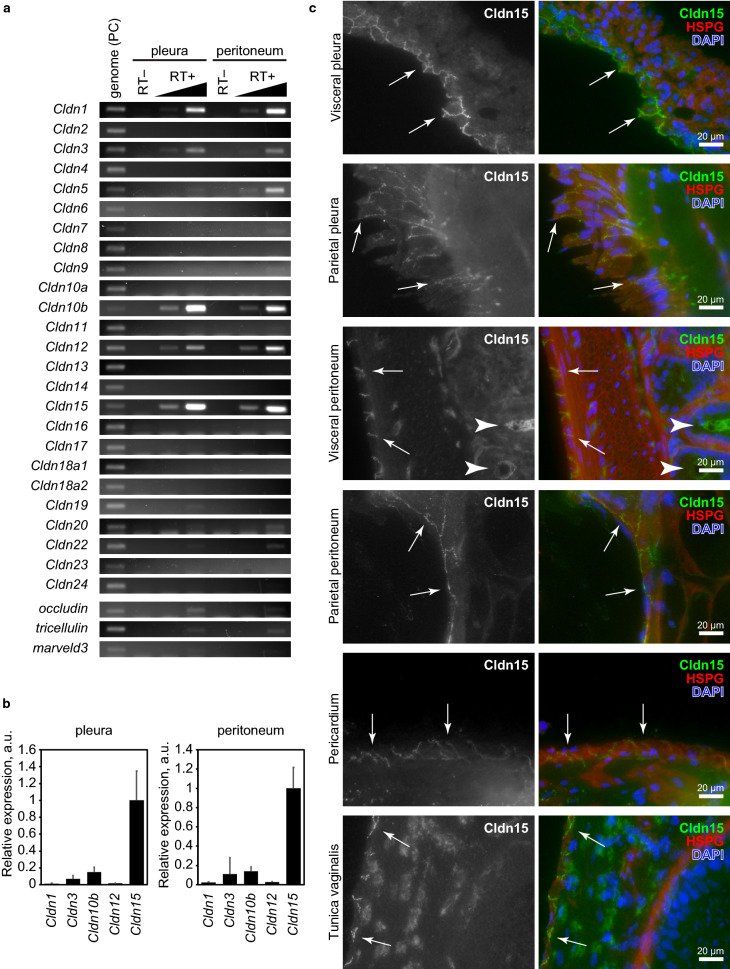
Claudin-15 is the major constituent of mesothelial tight junctions. **a** RT-PCR of mouse pleura and peritoneum using specific primers for claudins. Note that claudin-1, -5, -10b, -12 and -15 are expressed in the mesothelial tissues and that tight junction markers occludin and tricellulin are also expressed. Genomic DNA and samples prepared without reverse transcriptase were used as positive and negative controls, respectively. **b** Real-time RT-PCR of claudins. The relative expression levels were calculated using purified DNA fragments of each claudin as a standard. Note that claudin-15 is most abundantly expressed in both the pleura and peritoneum samples. **c** Immunofluorescence staining of mesothelial tissues. Visceral and parietal pleura, visceral and parietal peritoneum, pericardium, and tunica vaginalis were stained for claudin-15 (green), HSPG (basement membrane marker, red) and DAPI (DNA, blue). Note that claudin-15 is localized at cell–cell junctions of mesothelial cells.

To identify claudin(s) expressed in mesothelial cells, we conducted a comprehensive RT-PCR screening using mouse pleura and peritoneum and specific primer sets for all claudins (Fig. 1a). Among the 24 claudin members, only Cldn1, Cldn3, Cldn10b, Cldn12, and Cldn15 were detected in pleura tissue. In peritoneum tissue, Cldn5 was also detected, which is probably caused by a contamination of mesentery blood vessels. To directly compare the expression levels of these five claudin subtypes, we conducted real-time PCR using DNA fragments with titrated concentrations as standards, and calculated the expression levels of each claudin (Fig. 1b). As a result, Cldn15 was most abundantly expressed in both the pleura and peritoneum tissues. To confirm that Cldn15 is expressed in mesothelial cells, we immunostained mouse pleura, peritoneum, pericardium, and tunica vaginalis (Fig. 1c). In all mesothelial tissues examined, Cldn15 was detected at the cell–cell junctions of the mesothelial cells, which is consistent with the fact that claudin is a component of tight junctions. These data indicate that Cldn15 is the major claudin expressed in mesothelial cells in mice.

### Establishment of novel rat anti-human CLDN15 antibodies

**Figure 2:**
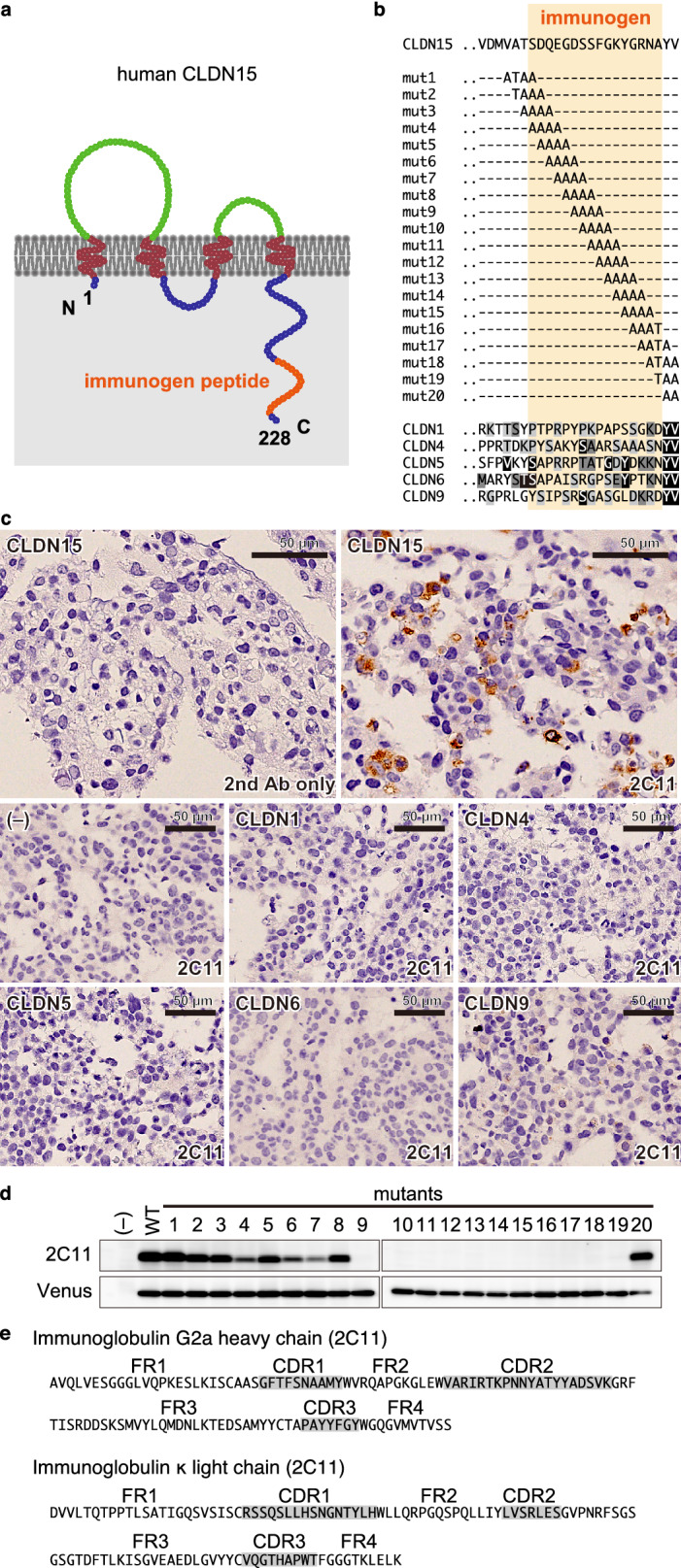
Establishment of a novel anti-CLDN15 mAb suitable for IHCs. **a** Schematic representation of the domain structure of human CLDN15 protein. The region corresponding to the peptide used for the immunization is shown in orange. **b** Amino-acid sequence of the cytoplasmic tail of CLDN15 and alanine mutants used in this study. Alignment with other CLDNs is also shown. **c** IHC of HEK293T cells expressing CLDNs. Note that 2C11 specifically stains only CLDN15-expressing cells. **d** Epitope analysis of 2C11. Note that 2C11 signal is lost in mutants 9–19, indicating that the epitope is ^219^FGKYGRNA^226^ of CLDN15. **e** Amino-acid sequences of CDRs of 2C11.

To examine the expression of CLDN15 in human specimens, we newly generated mAbs that are highly specific for CLDN15. Since the claudin proteins have high amino-acid sequence homology among family members, some antibodies against a claudin cross-react with other claudin members. We chose an immunogen peptide specific to CLDN15 in order to avoid any cross-reaction with other claudin members (Fig. 2a). We decided on amino-acid sequences of the cytoplasmic tails of human CLDN15 without homology with five representative claudins (Fig. 2b). We immunized rats with the peptide and screened for hybridomas producing an antibody applicable for immunohistochemistry (IHC). We then isolated an antibody clone (2C11), which specifically recognizes CLDN15 by IHC of formalin-fixed paraffin-embedded (FFPE) samples of CLDN15-overexpressing HEK293T cells (Fig. 2c). We also detected endogenous CLDN15 using 2C11 by IHC of an FFPE sample of human colon adenocarcinoma cell line Caco-2 cells (see Supplemenatry Fig. S1 online). To determine the epitope of this antibody, we generated 20 mutants of CLDN15 in which four amino acids in the immunogen region are replaced with alanine or threonine (Fig. 2b). Using HEK293T cell lysates expressing these mutants, we identified amino acids 219–226 (FGKYGRNA) as the epitope of 2C11 (Fig. 2d). We also isolated another CLDN15-specific clone, 3H11, which recognizes the region of amino acids 216–223 (DSSFGKYG) (see Supplementary Fig. S2 online). Furthermore, we determined the amino-acid sequences of the complementary determining region (CDR) of the 2C11 and 3H11 antibodies (Fig. 2e and Supplementary Fig. S2c online).

### CLDN15 protein is expressed in MPM tissues

**Figure 3:**
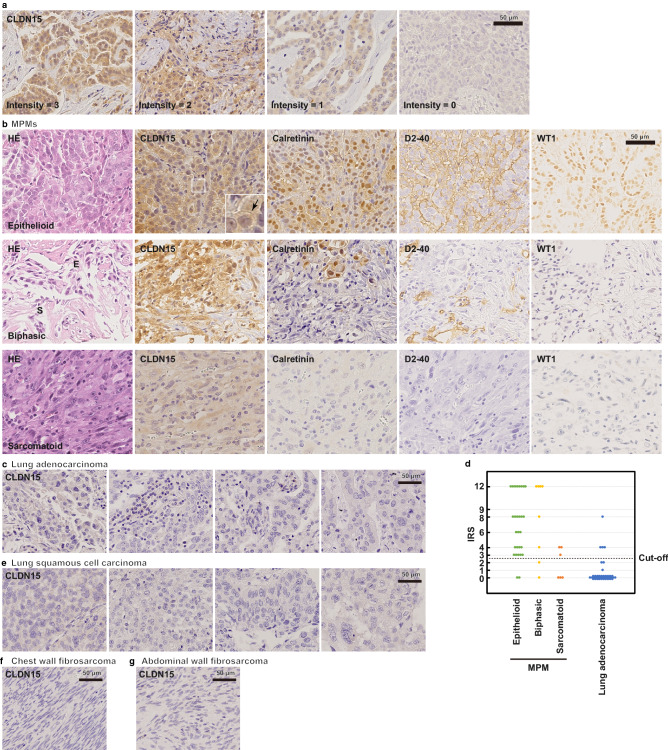
CLDN15 is expressed in MPM tissues. **a** Typical staining patterns of anti-CLDN15 (2C11) mAb exhibiting strong to weak staining intensities. Scale bar, 50 µm. **b** IHC of epithelioid-, biphasic- and sarcomatoid-type MPMs using anti-CLDN15 (2C11), anti-calretinin, anti-D2-40 and anti-WT1 antibodies. “E” and “S” in the HE staining panel of the biphasic-type MPM indicate the epitheliod-type and sarcomatoid-type cell regions, respectively. **c** IHC of lung adenocarcinomas using anti-CLDN15 (2C11) mAb. **d** Distribution of IRS scores in MPMs and lung adenocarcinomas. **e**–**g** IHC of lung squamous cell carcinomas (**e**), chest wall fibrosarcoma (**f**) and abdominal wall fibrosarcoma (**g**) using anti-CLDN15 (2C11) mAb.

Next, we examined FFPE samples of MPMs using the newly established anti-CLDN15 antibody 2C11. We collected tissue samples of 42 cases diagnosed as MPM. The background of the patients, including age, gender, histological type, asbestos exposure, stage (UICC, 8th^26^), and overall survival, is shown in Table 1. We examined the expression of CLDN15 in MPM tissue samples by immunohistochemistry using the 2C11 clone, and found that the membrane, as well as the cytoplasm, of only the tumor cells are positive for CLDN15 (Fig. 3a, b). We semi-quantified the expression levels of CLDN15 using an immunoreactive score (IRS) method. We classified the staining intensity (I) of the CLDN15 signal into four categories (Fig. 3a), and the proportion (P) of the staining-positive areas into five categories, and calculated the IRS (I × P) (for details, see the “Methods”). Cells with either membranous or cytoplasmic stain were considered as CLDN15-positive. When the cut-off value of the IRS was set to 3, 83% of the MPM samples were positive for CLDN15 (Fig. 3d; Table 2). A similar staining pattern was obtained using another anti-CLDN15 clone, 3H11 (see Supplementary Fig. S2 online). The positive ratio was equal to or greater than the other positive diagnostic markers for MPMs, calretinin (81%), WT-1 (50%), and D2-40 (81%). The positive ratio of CLDN15 was significantly higher than that of WT-1 (*p* = 0.0057). The positive ratio was different in each histological type. In the epithelioid type, the positive ratio for CLDN15 was 93%, which was similar to or even greater than the other markers (Calretinin, 93%; WT-1, 50%; D2-40, 86%). Again, the difference between CLDN15 and WT-1 was significant (*p* = 0.0022). In the biphasic type, CLDN15 was positive in 75%, which was similar to the other markers (Calretinin, 75%; WT-1, 63%; D2-40, 75%). The intensity and proportion in the epithelioid-type cell region was higher than those in the sarcomatoid-type cell region (Fig. 3b). In the sarcomatoid type, CLDN15 was positive in 50% of cases, which is comparable with the other markers (Calretinin, 33%; WT-1, 33%; D2-40, 67%) (Table 2). There was no significant difference in the sensitivity between any two markers in the biphasic and sarcomatoid types, although we could not rule out the possibility that there is a difference in the sensitivity among these markers since the statistical power was relatively low (0.20–0.28) due to small case numbers in these MPM types. In comparison to epithelioid-type MPMs, CLDN15 was significantly less positive in biphasic/sarcomatoid-type MPMs (*p* = 0.028).

**Table 1:** Clinicopathological features of MPM patients.

Number of patients	N = 42
Age (years)	
Median	65.5
Range	21–86
Gender [n (%)]	
Male	38 (90.5%)
Female	4 (9.5%)
Histological type [n (%)]	
Epithelioid	28 (66.7%)
Biphasic	8 (19.0%)
Sarcomatoid	6 (14.3%)
Asbestos exposure [n (%)]	
Exposed	27 (64.3%)
Non-exposed	15 (35.7%)
Stage (UICC 8th)	
I	28 (66.7%)
II	2 (4.8%)
III	8 (19.0%)
IV	4 (9.5%)
Survival (months)	
Median	9
Range	2–64

**Table 2:** Expression of CLDN15 and other markers for MPM in three types of MPMs and lung adenocarcinoma tissues.

	CLDN15	Calretinin	WT-1	D2-40	Total	
Epithelioid-type MPM	26 (93%)**	26 (93%)**	14 (50%)	24 (86%)*	28	
Biphasic-type MPM	6 (75%)	6 (75%)	5 (63%)	6 (75%)	8	
Sarcomatoid-type MPM	3 (50%)	2 (33%)	2 (33%)	4 (67%)	6	
Total MPM	35 (83%)*	34 (81%)*	21 (50%)	34 (81%)*	42	
Lung adenocarcinoma	4/50	4/40	1/40	0/40		
Specificity	92%	90%	98%	100%		

	CL15 or Cal	CL15 or WT1	CL15 or D2-40	Cal or WT1	Cal or D2-40	WT1 or D2-40
Epithelioid-type MPM	28 (100%)	27 (96%)	28 (100%)	27 (96%)	27 (96%)	26 (93%)
Biphasic-type MPM	7 (88%)	7 (88%)	7 (88%)	7 (88%)	8 (100%)	8 (100%)
Sarcomatoid-type MPM	4 (67%)	4 (67%)	5 (83%)	3 (50%)	5 (83%)	5 (83%)
Total MPM	39 (93%)	38 (90%)	40 (95%)	37 (88%)	40 (95%)	39 (93%)
Lung adenocarcinoma	7/40	5/40	4/40	5/40	4/40	1/40
Specificity	83%	88%	90%	88%	90%	98%

	CL15 & Cal	CL15 & WT1	CL15 & D2-40	Cal & WT1	Cal & D2-40	WT1 & D2-40
Epithelioid-type MPM	24 (71%)	13 (46%)^†^	22 (79%)	13 (46%)^†^	23 (82%)^‡^	12 (43%)^†^
Biphasic-type MPM	5 (63%)	4 (50%)	5 (63%)	4 (50%)	4 (50%)	3 (38%)
Sarcomatoid-type MPM	1 (17%)	1 (17%)	2 (33%)	1 (17%)	1 (17%)	1 (17%)
Total MPM	30 (71%)	18 (43%)	29 (69%)^‡^	18 (43%)	28 (67%)	16 (38%)^†^
Lung adenocarcinoma	1/40	0/40	0/40	0/40	0/40	0/40
Specificity	98%	100%	100%	100%	100%	100%

*p < 0.05; **p < 0.005 higher than the sensitivity of WT-1; ^†^*p* < 0.05 lower than CL15&Cal; ^‡^*p* < 0.05 higher than WT1&D2-40. Other pairs were not significantly different.

Since diagnostic markers are often applied in combination in clinical practice, we examined the sensitivity of two-marker panels (Table 2). Combination of two markers increased the sensitivity. For example, 93% of the samples were positive for CLDN15 or Calretinin (Table 2, middle). There were no statistically significant differences in the sensitivity between any two-marker panels, which is probably due to small sample size and low statistical power (0.20–0.35). 71% of samples were positive for both CLDN15 and Calretinin, indicating that the positivity of CLDN15 and Calretinin does not completely overlap (Table 2, bottom).

### CLDN15 is rarely expressed in other cancers

Since it is clinically important to differentiate lung adenocarcinomas from MPMs, we immunostained lung adenocarcinoma sections for CLDN15 and other positive markers. In the present study, 4/50 (8%) cases were positive for CLDN15, and the specificity was comparable with other markers (Calretinin, 4/40 [10%]: WT-1, 1/40 [2%]; D2-40, 0/40 [0%]) (Fig. 3c; Table 2). In the two-marker panels, the specificity was 83–98% and only one lung adenocarcinoma sample expressed two markers simultaneously. We also investigated the expression of CLDN15 on non-mesothelioma tumors by using tissue microarrays of 24 primary tumors. CLDN15 was expressed in only 12/327 (4%) cases (Fig. 3e–g; Table 3).

**Table 3:** CLDN15 expression in the tissue microarray of non MPM primary tumors.

Organ	Malignant tumor	CLDN15	Specificity (%)
Cerebrum	Astrocytoma	0/10	100
	Glioblastoma	1/10	90
Thyroid gland	Papillary carcinoma	0/10	100
	Follicular cell carcinoma	1/10	90
Lung	Squamous cell carcinoma	0/11	100
	Large cell carcinoma	1/12	92
	Small cell carcinoma	0/12	100
Breast	Invasive ductal carcinoma	0/19	100
Esophagus	Adenocarcinoma	0/10	100
	Squamous cell carcinoma	0/10	100
Stomach	Adenocarcinoma	0/20	100
Liver	Hepatocellular carcinoma	1/17	94
Pancreas	Duct adenocarcinoma	0/20	100
Colon	Adenocarcinoma	0/11	100
	Mucinous adenocarcinoma	0/7	100
Kidney	Clear cell carcinoma	6/20	70
Bladder	Invasive urothelial carcinoma	0/19	100
Prostate	Adenocarcinoma	1/20	95
Testis	Seminoma	0/10	100
	Embryonal carcinoma	0/10	100
Ovary	High grade serous carcinoma	1/9	89
	Mucinous adenocarcinoma	0/10	100
Cervix	Squamous cell carcinoma	0/20	100
Uterus	Endometrioid adenocarcinoma	0/20	100
Total		12/327	96.3

## Discussion

Although several reports suggested that the expression level of the *CLDN15* transcript is elevated in MPM tissues^22–24,27^, to date there has been no report examining the protein expression of CLDN15 in MPMs. In this study, we showed that claudin-15 is the most abundantly expressed claudin in mesothelial tissues and demonstrated that CLDN15 protein is also detected at a high level in MPM tissues using newly established anti-human CLDN15 mAbs. Furthermore, CLDN15 was negative in most tumors examined, including lung adenocarcinomas. Thus, we propose that CLDN15 is a promising positive marker for MPMs in clinical applications.

IHC is the most useful and standard method for the diagnosis of MPMs. The most required property as a positive marker is its high sensitivity. In this respect, the positive rate of CLDN15 in MPMs is 83%, which is equivalent to the conventional typical positive markers, such as Calretinin (81%), WT-1 (50%), and D2-40 (81%). Although Calretinin is widely used as a positive marker for MPMs, the proportion of the staining-positive area for Calretinin is not always high^10^. On the other hand, CLDN15 is often positive over a large portion of cancerous tissue, which is beneficial for the diagnosis of small samples, such as biopsy tissues. The positive rate of CLDN15 was as high as 93% in the epithelioid-type MPMs, while the sensitivity of CLDN15 in sarcomatoid-type MPMs was not so high (50%). This may reflect the reduced expression of CLDN15 in the less differentiated (more advanced) sarcomatoid types. Since the positivity for CLDN15 and Calretinin does not completely match, some cases are CLDN15-positive and Calretinin-negative, and vice versa. This indicates that the combinational application of CLDN15 and Calretinin (and other markers) can achieve much higher sensitivity (93% in our samples).

In addition to sensitivity, the immunohistochemical markers should have high specificity to distinguish MPMs from other types of cancers. An important cancer to differentiate from MPM is lung adenocarcinoma; the false-positive rate of CLDN15 in lung adenocarcinoma is 8%, which is comparable with other MPM-positive markers, such as calretinin (10%). In lung squamous cell carcinomas, which are another cancer to be distinguished from MPMs, we found no false positives for CLDN15 (n = 11). Since cancers of other organs may metastasize to the pleura, we also immunostained non-mesothelioma tumors other than lung cancers. CLDN15 was positive in only 12 out of 327 cases, although this result should be further validated in a larger cohort. Therefore, we concluded that CLDN15 has a high specificity comparable to current positive markers in clinical use.

The differential diagnosis of MPMs has been challenging because of the histological variety and morphological similarities with other cancers. Current diagnosis relies on immunostaining using a combination of multiple positive and negative markers, which have varied specificity and sensitivity. Since an increasing number of MPM cases is predicted in the upcoming decades, the establishment of a novel strategy using a differential combination of diagnostic markers is anticipated.

Although the anti-CLDN15 antibodies we established have high sensitivity and specificity (Fig. 3; Tables 2, 3), the CLDN15 signal was at the cytoplasm as well as membranes, which is in contrast to the exclusive membrane staining patterns of many other anti-CLDN antibodies. This may be due to internalization of CLDN15 into the cytoplasm when overexpressed. Indeed, overexpressed CLDN15 is localized at the cytoplasm in HEK293T cells (Fig. 2c). A similar change in claudin localization has been reported for CLDN1 in breast cancer, colon cancer and tongue carcinoma; the localization of CLDN1 changes from the membrane to the cytoplasm as the malignancy progresses^28,29^.

As for treatment strategies, there are only few effective treatments for unresectable MPMs. Platinum-based chemotherapy is the current first-line standard therapy for unresectable MPMs^30–32^. There was no recommended therapy for MPMs that have progressed after first-line chemotherapy. Thus, the development of new therapeutic strategies has been ongoing for long time. In recent years, several clinical trials have shown the effectiveness of immune checkpoint inhibitors such as the anti-programmed cell death 1 (PD-1) antibody against MPMs^33–35^. Therefore, it has become the standard therapy to administer anti-PD-1 antibody for patients with relapsed MPM who have received platinum-based chemotherapy^36^. In addition, various gene mutations were recently identified in MPMs, but molecular-targeted therapeutic agents such as those for non-small cell lung cancer have not yet been developed^37^.

The results of this study suggest that CLDN15 is a therapeutic target for MPMs. Since CLDN15 is expressed on the cell surfaces of MPM cells specifically, it would be a potential target for anti-cancer drugs. There may be several strategies for developing a new treatment of MPMs targeting CLDN15, one of which is developing antibodies against the extracellular region of CLDN15 with antibody-dependent cellular cytotoxicity^38^. Another strategy is an antibody–drug conjugate^39^. Radioimmunotherapy using radiolabeled antibody is also considerable because MPMs are regarded as radiosensitive^40–42^. In addition, chimeric antigen receptor (CAR)-T cell therapy is also attractive. CAR-T approach targeting the extracellular domain of another claudin (CLDN6) has been shown to successfully mediate a complete regression of CLDN6-expressing human tumor cells xenografted in immunodeficient mice^43^. However, it should be noted that CLDN15 constitutes the tight junctions of the normal intestinal epithelial cells and renal endothelial cells^44,45^, which might bear the risk of off-target toxicity in these approaches.

Because MPMs are rare tumors compared with lung cancers, it is difficult to find a large number of patients to enroll. Therefore, future studies with larger cohorts would further validate the results of the current study.

In conclusion, we revealed that IHC of CLDN15 has high sensitivity and specificity for MPM tissues in human. The results strongly support the potency of CLDN15 as a positive marker for the pathological diagnosis of MPM in clinical application.

## Methods

### Ethics approval and consent to participate

This study was reviewed by the Ethics Committee of Fukushima Medical University, approved by the president of the university (approval number 2512). The requirement for patients' informed consent was waived and the patients were given the right to opt-out from the research, which was stated on the website of Fukushima Medical University. This study was carried out in accordance with the Declaration of Helsinki and in compliance with the Japanese Ethical Guidelines for Medical and Health Research involving Human Subjects.

### Animal experiments

The experiments conducted using animals strictly adhered to the compliance standards of the Japanese Guidelines for Proper Conduct of Animals Experiments and ARRIVE guidelines. The protocols of the animal experiments (approval numbers 29098, 2019023, and 30112) were reviewed by the Fukushima Medical University Animal Care and Use Committee, and were approved by the president of the university.

### Patients and histological samples

The tissues were obtained from patients who were diagnosed as having malignant mesothelioma or lung adenocarcinoma between 2003 and 2019 at Fukushima Medical University Hospital and its partner hospitals. The multi organ tumor tissue array slide (MC6163) was purchased from US Biomax Inc (Derwood, MD, USA).

### Immunohistochemical staining of formalin-fixed paraffin-embedded samples

All the tissues of malignant pleural mesothelioma and lung adenocarcinoma were fixed with 10% formalin, embedded in paraffin, and subjected to Hematoxylin–Eosin (HE) staining or immunohistochemical staining. The sections of the samples were deparaffinized with 100% xylene and washed with 100% ethanol. The sections were then treated with 0.3% hydrogen peroxide-containing methanol at room temperature (RT) for 20 min to inactivate the endogenous peroxidase followed by 0.1% semicarbazide hydrochloride (#192–00372; Fujifilm-WAKO, Osaka, Japan) in distilled water for 1 h at RT and Immunosaver (Nissin EM, Tokyo Japan) at 70 °C for 16 h. Blocking was performed with 5% non-fat dry milk (#0646869; Morinaga Milk Industry) in phosphate-buffered saline (PBS). The sections were treated with primary antibody in PBS containing 2% bovine serum albumin (BSA) at 4ºC overnight. The secondary antibody reaction was carried out at RT for 30 min. Then, the sections were developed with 3,3′-diaminobenzidine (DAB) solution (50 mM Tris buffer [pH 7.5], 0.02% [w/w] DAB, 30%, 0.005% hydrogen peroxide) for 20 min at RT, and counter-stained with hematoxylin. Samples were dehydrated with 100% ethanol and then 100% xylene, and embedded. The samples were observed using an optical phase contrast microscope (OLYMPUS BX61, OLYMPUS, Tokyo, Japan), and images were taken with a DP controller (OLYMPUS).

### Histological evaluation

After masking the patient background, two pathologists and one thoracic surgeon semi-quantified the CLDN15 protein expression using an immunoreactive score (IRS; partially modified from Remmele W et al.^46^) method. We classified the staining intensity (I) of the CLDN15 signal into four categories (0 [negative], 1 [weak], 2 [moderate], and 3 [strong]) and the proportion (P) of staining-positive areas in the tumor into five categories (0 [< 1%], 1 [1–10%], 2 [11–30%], 3 [31–50%], and 4 [50% <]). Then, “I” and “P” were multiplied to obtain IRS (IRS = I × P).

### Statistical analysis

Statistical significance of differences was evaluated by the Fisher's Exact test with Bonferroni's correction. The *p*-value was calculated by Excel software and *p* < 0.05 was considered to be statistically significant. The statistical power was calculated by G*Power software using the phi (φ) coefficient as the effect size.

## Data availability

The datasets and monoclonal antibodies used in the current study are available from the corresponding author on reasonable request.

## Supplementary Information

Supplementary Information.

## References

[1] Bianchi C, Bianchi T (2007). Malignant mesothelioma: global incidence and relationship with asbestos. Ind. Health.

[2] Robinson BWS, Lake RA (2005). Advances in malignant mesothelioma. N. Engl. J. Med..

[3] Gemba K (2012). National survey of malignant mesothelioma and asbestos exposure in Japan. Cancer Sci..

[4] Wagner JC, Sleggs CA, Marchand P (1960). Diffuse pleural mesothelioma and asbestos exposure in the North Western Cape Province. Br. J. Ind. Med..

[5] Yap TA, Aerts JG, Popat S, Fennell DA (2017). Novel insights into mesothelioma biology and implications for therapy. Nat Rev Cancer.

[6] Clayson, H., Seymour, J. & Noble, B. Mesothelioma from the patient's perspective. *Hematol. Oncol. Clin. North Am.* **19**, 1175–1190, viii. 10.1016/j.hoc.2005.09.003 (2005). 10.1016/j.hoc.2005.09.003 16325130

[7] Taioli E (2015). Determinants of survival in malignant pleural mesothelioma: a surveillance, epidemiology, and end results (SEER) study of 14,228 patients. PLoS ONE.

[8] Myers, D. J. & Wallen, J. M. Lung Adenocarcinoma. *StatPearls* (StatPearls Publishing Copyright© 2021, StatPearls Publishing LLC., 2021).

[9] Travis WD, Brambilla E, Burke AP, Marx A, Nicholson AG (2015). Introduction to the 2015 World Health Organization classification of tumors of the lung, pleura, thymus, and heart. J. Thorac. Oncol..

[10] Husain AN (2018). Guidelines for pathologic diagnosis of malignant mesothelioma 2017 update of the consensus statement From the International Mesothelioma Interest Group. Arch. Pathol. Lab Med..

[11] Chapel DB, Schulte JJ, Husain AN, Krausz T (2020). Application of immunohistochemistry in diagnosis and management of malignant mesothelioma. Transl. Lung Cancer Res..

[12] Washimi K (2012). Specific expression of human intelectin-1 in malignant pleural mesothelioma and gastrointestinal goblet cells. PLoS ONE.

[13] Tsuji S (2017). HEG1 is a novel mucin-like membrane protein that serves as a diagnostic and therapeutic target for malignant mesothelioma. Sci. Rep..

[14] Oliveira SS, Morgado-Díaz JA (2007). Claudins: multifunctional players in epithelial tight junctions and their role in cancer. Cell Mol. Life Sci..

[15] Osanai M, Takasawa A, Murata M, Sawada N (2017). Claudins in cancer: bench to bedside. Pflugers Arch..

[16] Tabaries S, Siegel PM (2017). The role of claudins in cancer metastasis. Oncogene.

[17] Lanigan F (2009). Increased claudin-4 expression is associated with poor prognosis and high tumour grade in breast cancer. Int. J. Cancer.

[18] Ouban A (2018). Claudin-1 role in colon cancer: An update and a review. Histol. Histopathol..

[19] Singh AB, Dhawan P (2015). Claudins and cancer: Fall of the soldiers entrusted to protect the gate and keep the barrier intact. Semin. Cell Dev. Biol..

[20] Markov AG (2011). Tight junction proteins contribute to barrier properties in human pleura. Respir. Physiol. Neurobiol..

[21] Blum Y (2019). Dissecting heterogeneity in malignant pleural mesothelioma through histo-molecular gradients for clinical applications. Nat. Commun..

[22] Bueno R (2016). Comprehensive genomic analysis of malignant pleural mesothelioma identifies recurrent mutations, gene fusions and splicing alterations. Nat. Genet..

[23] Gordon GJ (2005). Identification of novel candidate oncogenes and tumor suppressors in malignant pleural mesothelioma using large-scale transcriptional profiling. Am. J. Pathol..

[24] Rouka E (2017). Transcriptomic Analysis of the claudin interactome in malignant pleural mesothelioma: evaluation of the effect of disease phenotype, asbestos exposure, and CDKN2A deletion status. Front. Physiol..

[25] Inai K (2008). Pathology of mesothelioma. Environ. Health Prev. Med..

[26] Berzenji L, Van Schil PE, Carp L (2018). The eighth TNM classification for malignant pleural mesothelioma. Transl Lung Cancer Res.

[27] Kanamori-Katayama M (2011). LRRN4 and UPK3B are markers of primary mesothelial cells. PLoS ONE.

[28] Bhat AA (2020). Claudin-1, a double-edged sword in cancer. Int. J. Mol. Sci..

[29] Yamamoto D (2020). Intracellular claudin-1 at the invasive front of tongue squamous cell carcinoma is associated with lymph node metastasis. Cancer Sci..

[30] Vogelzang NJ (2003). Phase III study of pemetrexed in combination with cisplatin versus cisplatin alone in patients with malignant pleural mesothelioma. J. Clin. Oncol..

[31] Ceresoli GL (2006). Phase II study of pemetrexed plus carboplatin in malignant pleural mesothelioma. J. Clin. Oncol..

[32] Castagneto B (2008). Phase II study of pemetrexed in combination with carboplatin in patients with malignant pleural mesothelioma (MPM). Ann. Oncol..

[33] Okada M (2019). Clinical efficacy and safety of nivolumab: results of a multicenter, open-label, single-arm, Japanese phase II study in malignant pleural mesothelioma (MERIT). Clin. Cancer Res..

[34] Quispel-Janssen J (2018). Programmed death 1 blockade with nivolumab in patients with recurrent malignant pleural mesothelioma. J. Thorac. Oncol..

[35] Scherpereel A (2019). Nivolumab or nivolumab plus ipilimumab in patients with relapsed malignant pleural mesothelioma (IFCT-1501 MAPS2): a multicentre, open-label, randomised, non-comparative, phase 2 trial. Lancet Oncol..

[36] Ettinger, D. S. *NCCN Clinical Practice Guidelines in Malignant Pleural Mesothelioma*. https://www2.tri-kobe.org/nccn/guideline/lung/english/mpm.pdf (2019).

[37] Yoshikawa Y, Kuribayashi K, Minami T, Ohmuraya M, Kijima T (2020). Epigenetic Alterations and Biomarkers for Immune Checkpoint Inhibitors-Current Standards and Future Perspectives in Malignant Pleural Mesothelioma Treatment. Front Oncol.

[38] Nimmerjahn F, Ravetch JV (2008). Fcgamma receptors as regulators of immune responses. Nat. Rev. Immunol..

[39] Hamilton GS (2015). Antibody-drug conjugates for cancer therapy: The technological and regulatory challenges of developing drug-biologic hybrids. Biologicals.

[40] Witzig TE (2002). Treatment with ibritumomab tiuxetan radioimmunotherapy in patients with rituximab-refractory follicular non-Hodgkin's lymphoma. J. Clin. Oncol..

[41] Andersson H (2009). Intraperitoneal alpha-particle radioimmunotherapy of ovarian cancer patients: Pharmacokinetics and dosimetry of (211)At-MX35 F(ab')2–a phase I study. J. Nucl. Med..

[42] Kratochwil C (2016). 225Ac-PSMA-617 for PSMA-targeted α-radiation therapy of metastatic castration-resistant prostate cancer. J. Nucl. Med..

[43] Reinhard K (2020). An RNA vaccine drives expansion and efficacy of claudin-CAR-T cells against solid tumors. Science.

[44] Chiba H, Osanai M, Murata M, Kojima T, Sawada N (2008). Transmembrane proteins of tight junctions. Biochim. Biophys. Acta.

[45] Kiuchi-Saishin Y (2002). Differential expression patterns of claudins, tight junction membrane proteins, in mouse nephron segments. J. Am. Soc. Nephrol..

[46] Remmele W (1986). Comparative histological, histochemical, immunohistochemical and biochemical studies on oestrogen receptors, lectin receptors, and Barr bodies in human breast cancer. Virchows Arch A Pathol. Anat. Histopathol..

