# Supplementary material for: CLDN15 is a novel diagnostic marker for malignant pleural mesothelioma
Source: Sci Rep. 2021 Jun 15;11:12554. doi: 10.1038/s41598-021-91464-0 (PMC8206149; doi:10.1038/s41598-021-91464-0)

Supplementary information

CLDN15 is a novel diagnostic marker for malignant pleural mesothelioma

Masayuki Watanabe[1,2], Tomohito Higashi[2*], Kana Ozeki[2], Atsuko Y. Higashi[2], Kotaro Sugimoto[2], Hayato Mine[1], Hironori Takagi[1], Yuki Ozaki[1], Satoshi Muto[1], Naoyuki Okabe[1], Yuki Matsumura[1], Takeo Hasegawa[1], Yutaka Shio[1], Hiroyuki Suzuki[1], Hideki Chiba[2]

[1]Department of Chest Surgery, [2]Department of Basic Pathology, Graduate School of Medicine, Fukushima Medical University, Fukushima, Japan.

Caco-2 cells

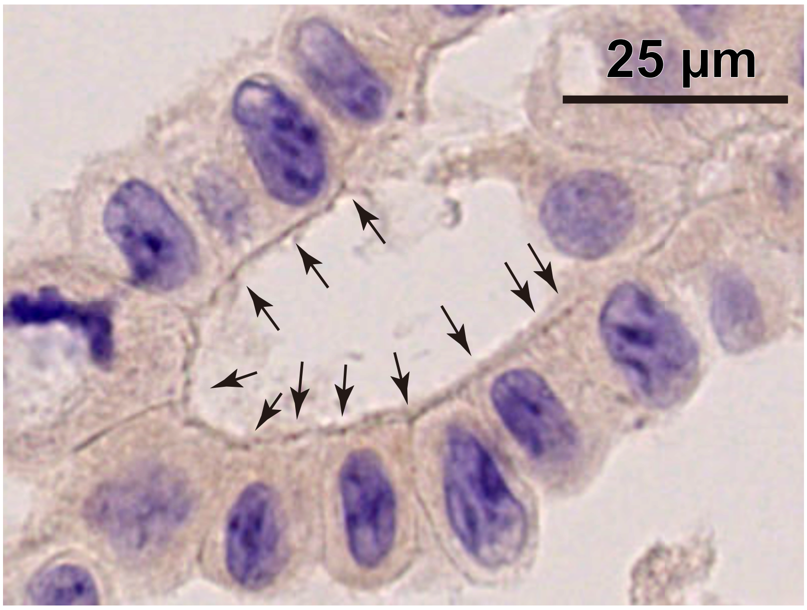

Figure S1. Anti-CLDN15 mAb 2C11 recognizes tight junctions in Caco-2 cells.
Cell sheet of Caco-2 cells were fixed, scraped off from the dish and embedded in the paraffin. The FFPE sample was stained with anti-CLDN15 (2C11). Note that the signal is detected on the cell membranes, especially at the apical tight junctions (arrows).

a

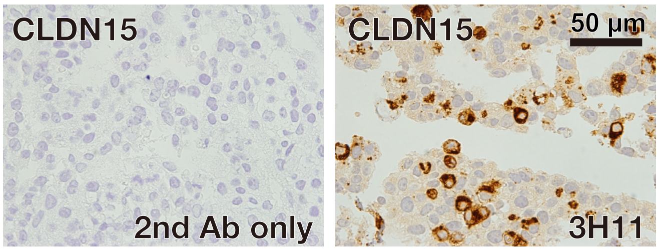

b

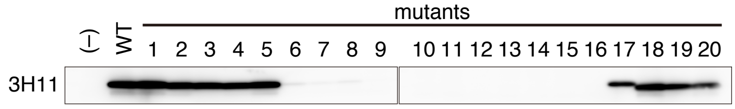

c

Immunoglobulin G2a heavy chain (3H11)

FR1 CDR1 FR2 CDR2 FR3 CDR3 FR4

KLSCAASGFTFSNYVMDWVRQAPKKGLEWVASISTGGGNTYYPDSVKGRFTISRDNAESTLYLQMNSLRSEDTATYYCAASYADYWGQGVMVTVSSAETTAPSV

Immunoglobulin κ light chain (3H11)

FR1 CDR1 FR2 CDR2 FR3 CDR3 FR4

DVVLTQTPPTLSATIGQSVSISCRSSQSLLHSNGNTYLNWLLQRPGQPPQLLIYLVSRLESGVPNRFSGSGSGTDFTLKISGVEAEDLGVYYCVQSTHAPRTFGGGTKLELK

d

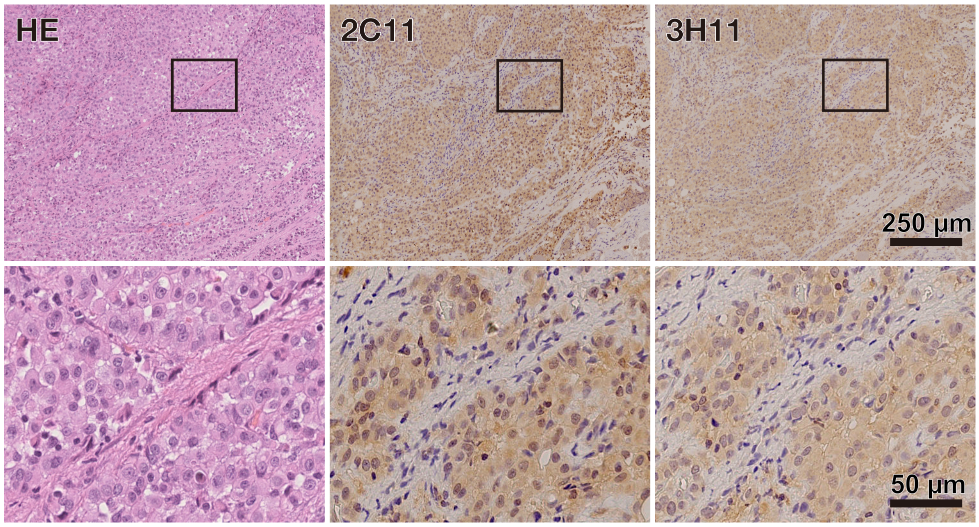

Figure S2. Anti-CLDN15 mAb clones 2C11 and 3H11 exhibit similar staining patterns.

a. IHC of CLDN15-expressing HEK293 cells using anti-CLDN15 (3H11).

b. Epitope analysis of 3H11 using immunoblotting of HEK293T cells expressing CLDN15 mutants shown in Figure 2. Note that 3H11 signal is lost in the mutant 6-16, which corresponds to ^{206}DSSFGKYG^{223} of CLDN15.

c. Amino-acid sequence of the CDRs of 3H11.

d. HE staining and IHC of an epithelioid-type MPM sample using 2C11 and 3H11 mAbs. Note that both clones give similar staining patterns despite their epitopes are different.

Supplementary Table 1. Primers used in the PCR, RT-PCR and real time RT-PCR experiments.

gene	forward	reverse
claudin-1	GAGGTGTCCTACTTTCCTGCTC	TCTCTTCCTTTGCCTCTGTCAC
claudin-2	CCAGGATTCTCGAGCTAAGGAC	CGAGTAGAAGTCCCGAAGGATG
claudin-3	AACTGCGTACAAGACGAGACG	GGTACTAAGGTGAGCAGAGCC
claudin-4	GATGCAGTGCAAGATGTACGAC	CCTACCACTGAGAGAAGCATCC
claudin-5	GTTGGAAATTCTGGGTCTGGTG	ACGATGTTGTGGTCCAGGAAG
claudin-6	TGTTGTCACCCTCCTCATTGTC	AGATGATGCCAGAGATGAGCAC
claudin-7	TCGAGCCTTAATGGTGGTGTC	GGCCTTCTTCGCTTTGTCATC
claudin-8	GGGTTGCCAATTCCATCATCAG	CAGCCTATGTAGAGGGCTTCTC
claudin-9	TAACCCACTGGTTGCGGAAG	GACACGTACAGCAGAGGAGC
claudin-10a	AGATCTCAGCTCTGGTGTGTG	GGTGGTCACTTTCCATTCGTTG
claudin-10b	AAATCGTCGCCTTCGTAGTCTC	TTGGCAAAATAAGTGGCTGTGG
claudin-11	TACGTGCAGGCTTGTAGAGC	GGATGCAGGGGAGAACTGTC
claudin-12	AACTGGCCAAGTGTCTGGTC	AGACCGGCTCAAACTTCCTG
claudin-13	CAAACAAGAGGCCATCAGCTTC	CTGGGTCTGTCTCATCATCTGG
claudin-14	CTAGGCTTCCTGCTTAGCTTCC	ACATTCCATCCACAGTCCCTTC
claudin-15	TGACCCTTTCAAACAGCTACTGG	CAGGTTCTCAAAGATGGTGTTGG
claudin-16	GATCTTCTTCAGTACGCTGCC	TTCACCATCCAACAGTCTGTCC
claudin-17	TCTTCGGTTTGGTTGGGACG	CTGCCGATGAAAGCTGACAC
claudin-18A1	TGTTCCAGTATGAAGGGCTCTG	GCCCAGGATGGTGAAGTATGG
claudin-18A2	TACCAAGGGCTATGGCGTTC	CCCAACAGGGTGAAGTAGCC
claudin-19	GGTCATATCCAGTCAGCACGAG	GTTACTGTCTCCAACCCGAGTG
claudin-20	TTTCCTTTGCTGGAGGAGTCTG	TTTCCGGAATGGTCAGATCCAG
claudin-22	TTAGTCTTCCGAACGGCAACG	CCATGGTCCAGTTCTCCATCTC
claudin-23	GTACTACAGCGACGGACAGC	TCGGGAATCCAACCTTGAGC
claudin-24	CAGCCTCGAGTCACTATGCAG	TACACTTTAGGCTGTACAGTTCCA
occludin	CATATTTGCCTGTGTGGCTTCC	AGGGTAGTTTAGGCTTCCTCCA
tricellulin	ACAGATGACGATCGAGAACGC	TCCAGCTCGTCAAACTTCCTC
marveld3	GGAACAGAGAACGGACCGAG	TCCAAGGCTCTTTGTTCGGATG
rat IgG-HC-RT	5'phos-CCAGGTGCTGGAG	
rat IgG-HC-1st	AACTCTGGAGCCCTGTCCAG	ACTGGCTCAGGGAAATAGCC
rat IgG-HC-2nd	CAGCTGTCCTGCAGTCTGG	GGATAGACAGATGGGGCTGTT
rat IgG-kappa-RT	5'phos-CATGCTGTACGTGC	
rat IgG-kappa-1st	GTGTCAAGTGGAAGATTGATG	CTGATGTCTCTGGGATAGAAG
rat IgG-kappa-2nd	GACAGTGTTACTGATCAGGAC	TACAGTTGGTGCAGCATCAGC

Supplementary methods

Preparation of claudin (CLDN)-expressing cell sample

Expression plasmid vectors encoding a claudin gene (CLDN1, CLDN4, CLDN5, CLDN6, CLDN9, and CLDN15), an internal ribosome entry site (IRES) and a fluorescent protein Venus under the control of the EF1α promoter were prepared. HEK293T cells were transfected with the plasmid using PEI-max (#24765-1; Polysciences, PA, USA). As a negative control, a plasmid vector containing no CLDN gene was used. In addition, 20 plasmids encoding CLDN15 mutants were prepared by PCR in which four amino acids were replaced with alanine or threonine within the immunogen region of the CLND15 gene. The cells were fixed with 10% formalin 48 h after transfection and embedded in paraffin to prepare a paraffin section of a CLDN-expressing cell mass. For immunoblotting, cells were lysed with a sodium dodecyl sulfate (SDS) sample buffer (60 mM Tris-HCl, pH 6.8, 2% SDS, 10% Glycerol, 0.25% Bromophenol blue, 5% 2-mercaptoehanol) 48 h after transfection, and were boiled for 5 min at 95ºC.

Production of rat anti-human CLDN15 mAbs

Monoclonal antibodies against human CLDN15 protein were prepared using the following method according to the rat iliac lymph node method [1]. A peptide corresponding to the 15 amino acids near the carboxy terminus of human CLDN15 (NH_2-Cys-SDQEGDSSFGKYGRNA-COOH) was synthesized (Eurofins Genomics, Tokyo, Japan) and dissolved in PBS to make a 5 mg/ml solution. Then, 2 mg of Imject Maleimide-activated mcKLH (#77606; Thermo Fisher Scientific, MA, USA) was dissolved in 200 µl of distilled water to prepare a 10

mg/ml KLH solution, mixed with the peptide solution, and incubated at RT for 2 h to make a KLH-conjugated peptide antigen. The antigen solution was dialyzed against 150 ml of PBS three times. Next, 250 µl of the antigen solution, 250 µl of PBS and 1 ml of Freund's complete adjuvant (F5881; Sigma-Aldrich, MO, USA) were mixed using two 2 ml Luer lock-type glass syringes and a connector to form an antigen emulsion. An 8-week-old female Wistar rat was anesthetized with isoflurane, and 100 µl of the antigen emulsion was subcutaneously injected into the footpad of each hind limb for immunization. For cell fusion, 5 g of PEG4000 (#109727; Merck millipore, Darmstadt, Germany) was autoclaved and mixed with 8 ml of Dulbecco's Modified Eagle's Medium (DMEM) (D5796; Sigma-Aldrich) and 0.4 ml of dimethyl sulfoxide (D2650; Sigma-Aldrich). Fourteen days after immunization, the rat was euthanized and both iliac lymph nodes were aseptically harvested. The lymph nodes were swollen by injecting a small amount of DMEM and then minced with scissors. The lymph node tissues were crushed and filtered on a 70-µm mesh cell strainer (BD Falcon). The collected lymphocytes were mixed with 1×10^7 SP2 cells, a mouse multiple myeloma-derived cell line, and centrifuged at 1280 rpm for 10 min. After the supernatant was removed and the cell pellet was thoroughly loosened, 1 ml of 50% PEG solution kept at 37ºC was slowly added with gentle mixing over 1 min to fuse the lymphocyte cells and SP2 cells. Two min later, 9 ml of DMEM kept at 37ºC was slowly added dropwise over 5 min to dilute the PEG, and the cells were centrifuged at 900 rpm for 5 min. The supernatant was removed and the cells were resuspended in 40 ml of hybridoma medium (78% GIT medium [#637-25715, Fujifilm WAKO], 2% HAT supplement [#21060017, Thermo Fisher Scientific], 10% BM CondiMed H1 Hybridoma

cloning supplement [#11088947001, Roche, Basel, Switzerland], 10% fetal bovine serum), seeded in four 96-well plates, and cultured at 37ºC in a CO_2 incubator. After 4 to 5 days of seeding, the medium was replaced with 200 µl of fresh hybridoma medium. After two days, hybridoma clones were screened by an enzyme-linked immunosorbent assay (ELISA) using the culture medium and antigen peptide. On the day before ELISA, 100 µl of antigen peptide solution (3 µg/ml in PBS) was added to each well of a 96-well ELISA plate (#655101, Greiner bio-one, Kremsmünster, Austria), and the plate was incubated overnight at 4ºC to coat the surface of the wells with the peptide. PBS alone was used as a negative control. Each well was blocked with a blocking solution (PBS containing 1% BSA) at 37ºC for 1 h, and then incubated with 50 µl of culture supernatant at 37ºC for 1 h. After washing the wells twice with PBS, 50 µl of the secondary antibody solution (horseradish peroxidase [HRP]-linked goat anti-Rat IgG [NA935; GE Healthcare, IL, USA; 1:2000 dilution]) was added to each well and incubated at 37ºC for 1 h. After washing three times with PBS, the plate was developed using 50 µl of 3,3',5,5'-tetramethylbenzidine (TMB) substrate (BioLegend, CA, USA). The reaction was stopped with 50 µl of 1M phosphoric acid, and then the absorbance at 450 nm was quantified. Two clones with a good titer (2C11 and 3H11) were selected.

Sequence analysis of the Complementarity-determining regions (CDRs) of the mAbs

Total RNAs were isolated from the anti-CLDN15 hybridoma clones using TRIzol reagent (#15596026; Thermo Fisher Scientific) according to the manufacturer's

instruction. The variable regions of immunoglobulin heavy and light chains were reverse-transcribed using 5'-Full RACE Core Set (#6122, Takara BIO, Kusatsu, Japan) and primers as shown in Supplementary Table 1. Then, the variable regions were further amplified with nested-PCR using GoTaq DNA polymerase (Promega, WI, USA) or PrimeSTAR GXL DNA Polymerase (Takara BIO) and primers shown in Supplementary Table 1 and cloned into pGEM-T-easy plasmid (#A137A, Promega). The DNA sequence of the plasmid was determined (Macrogen Japan) and the corresponding amino-acid sequences of the CDRs for heavy and light chains were identified.

Immunoblotting

Proteins in the HEK293T cell lysates were separated by SDS-polyacrylamide gel electrophoresis (PAGE) using a 12.5% gel (Fujifilm WAKO), and were transferred onto the PVDF membrane (Immobilon, Merck, Darmstadt, Germany). The membrane was blocked with 5% non-fat dried milk in tris-buffered saline (TBS) containing 0.1% Tween-20 (TBS-T) for 30 min at RT, and incubated with a primary mouse anti-GFP antibody (#598; 1:2000; MBL, Nagoya, Japan) diluted in TBS-T or culture supernatant of hybridomas overnight at 4ºC. After washing with TBS-T, the membrane was incubated with HRP-conjugated anti-Mouse IgG (GE Healthcare; NA931: 1:20000 dilution) or anti-Rat IgG (NA935; 1:5000 dilution) for 1 h at RT. The membrane was then incubated with ECL prime (GE Healthcare) and developed using LAS4000 (GE Healthcare). The gel images were processed using Photoshop (Adobe, CA, USA).

Immunofluorescence microscopy of frozen sections

Tissue sections of 8-μm thickness were fixed with 100% ethanol for 15 min at -20 ºC and washed with PBS three times. After blocking with 2% BSA in PBS, the sections were incubated with rabbit anti-claudin15 polyclonal antibody (pAb) (#18805; IBL, Gumma, Japan) and rat anti-heparan sulfate proteoglycan mAb (HSPG; MAB1948; Merck Millipore) for 30 min at RT. After washing with PBS, the sections were incubated with Alexa Fluor 488-conjugated donkey anti-rabbit IgG pAb (#711-545-150) and Cy3-conjugated donkey anti-rat IgGpAb (#712-165-153), and mounted with Fluoro-Gel II with DAPI (#17985-50, Electron Microscopy Sciences, PA, USA).

RT-PCR

Total RNAs were isolated from the mouse pleura and peritoneum using TRIzol reagent, and cDNA libraries were synthesized using the Primescript II 1st strand cDNA synthesis kit (#6210; Takara BIO). DNA fragments were amplified by PCR with GoTaq DNA polymerase using specific primers for each claudin (listed in Supplementary Table 1). The primer pairs were designed in the same exons, and the DNA fragments amplified from genome served as positive controls. Samples without reverse transcription served as negative controls. The images were recorded using LAS4000 and processed with Photoshop software.

Real-time RT-PCR

Real-time RT-PCR was performed with StepOne Real-Time PCR System (Applied Biosystems, CA, USA). The RT-PCR products were used as templates

for amplification using Thunderbird SYBR qPCR Mix (Toyobo, Osaka, Japan) and specific primers for each claudin (listed in Supplementary Table 1). The relative expression levels were standardized using the DNA fragments amplified from genome DNA, and were normalized to the expression level of *claudin-15*.

1. Kishiro, Y., Kagawa, M., Naito, I. & Sado, Y. A novel method of preparing rat-monoclonal antibody-producing hybridomas by using rat medial iliac lymph node cells. *Cell Struct Funct* **20**, 151-156 (1995).

Original unprocessed gel/blot images

Fig. 1a

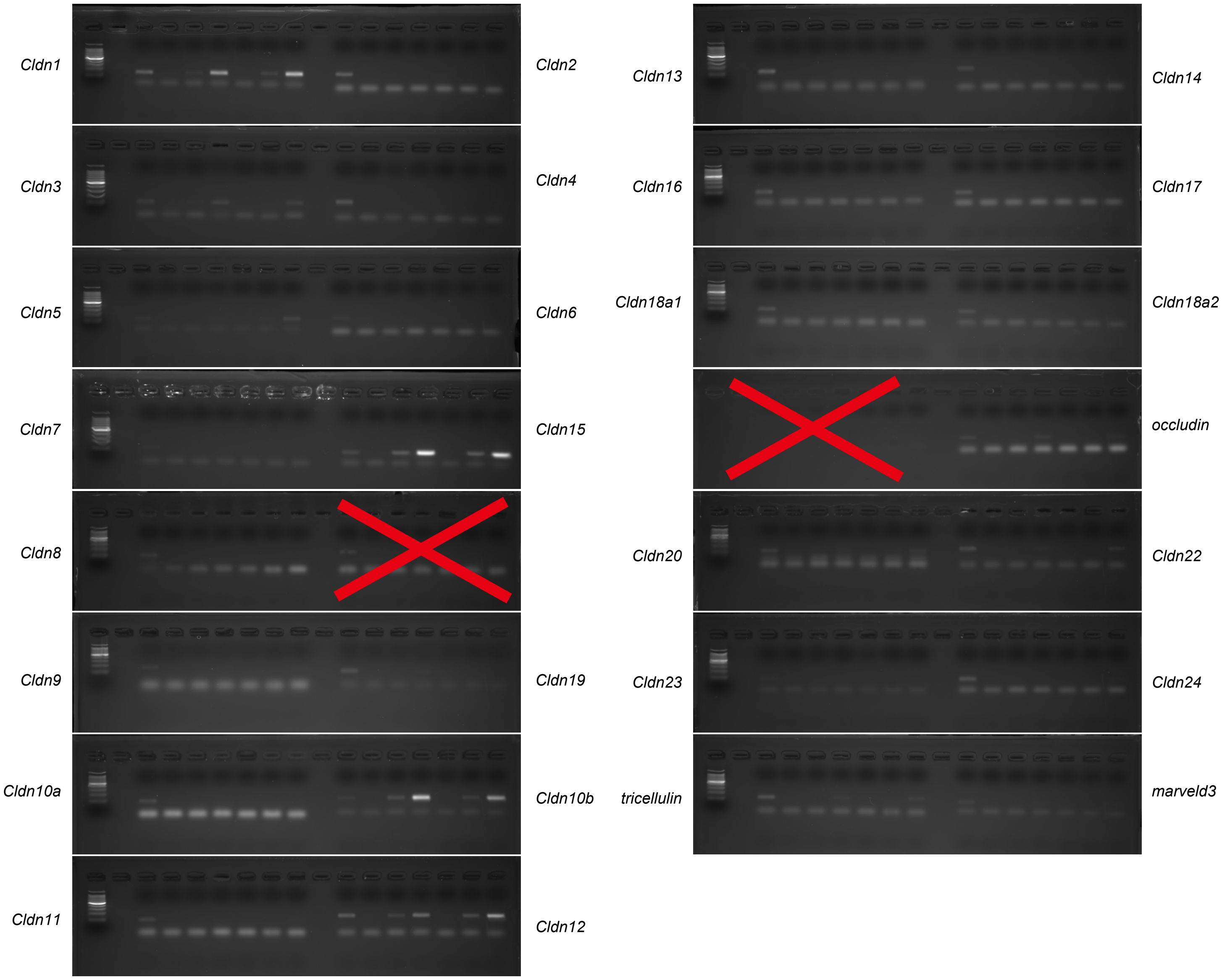

Fig. 2d

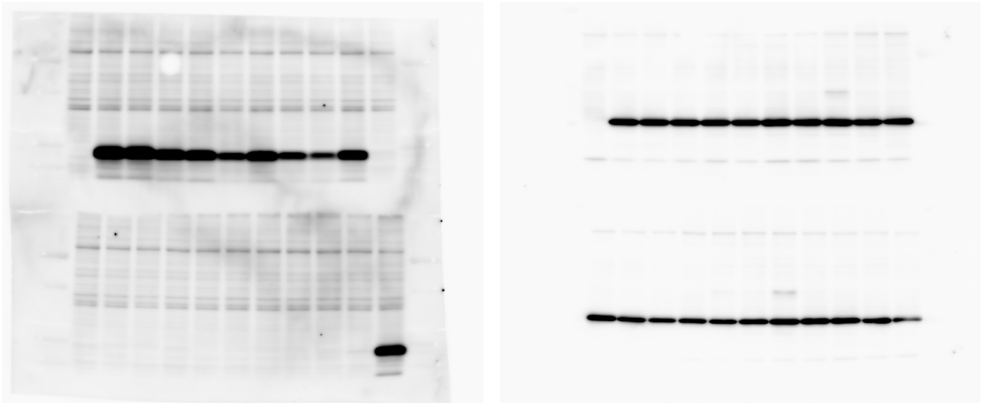

Fig. S2b

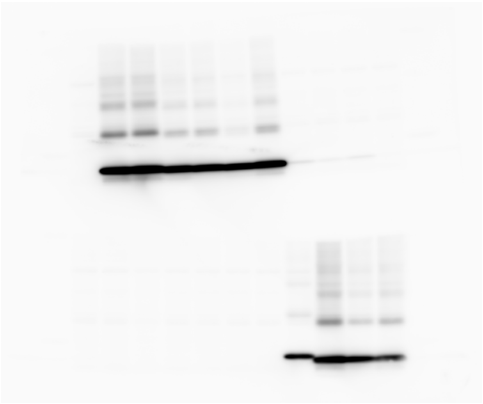

Supplement: Supplementary file 1 — Supplementary Information. [file 41598_2021_91464_MOESM1_ESM.pdf]
